# New Evidence for the Existence of Two Kiss/Kissr Systems in a Flatfish Species, the Turbot (*Scophthalmus maximus*), and Stimulatory Effects on Gonadotropin Gene Expression

**DOI:** 10.3389/fendo.2022.883608

**Published:** 2022-06-15

**Authors:** Chunyan Zhao, Bin Wang, Yifan Liu, Chengcheng Feng, Shihong Xu, Wenqi Wang, Qinghua Liu, Jun Li

**Affiliations:** ^1^School of Marine Science and Engineering, Qingdao Agricultural University, Qingdao, China; ^2^The Key Laboratory of Experimental Marine Biology, Centre for Ocean Mega-Science, Institute of Oceanology, Chinese Academy of Sciences, Qingdao, China; ^3^Laboratory for Marine Biology and Biotechnology, Pilot National Laboratory for Marine Science and Technology, Qingdao, China; ^4^Key Laboratory of Sustainable Development of Marine Fisheries, Ministry of Agriculture and Rural Affairs, Yellow Sea Fisheries Research Institute, Chinese Academy of Fishery Sciences, Qingdao, China; ^5^National Engineering Research Center for Marine Aquaculture, Zhejiang Ocean University, Zhoushan, China; ^6^Southern Marine Science and Engineering Guangdong Laboratory (Guangzhou), Guangzhou, China

**Keywords:** turbot, kisspeptin, kisspeptin receptor, reproduction, gonadal development

## Abstract

Seasonal reproduction is generally controlled by the hypothalamus-pituitary-gonadal (HPG) axis in fish. Previous studies have demonstrated that the kisspeptin (Kiss)/kisspeptin receptor (Kissr) system, a positive regulator of the HPG axis, mediates the responses to environmental cues. Turbot (*Scophthalmus maximus*), a representative species of Pleuronectiformes, is one of the most commercially important fish species cultured in Europe and North China. However, the mechanisms by which the Kiss/Kissr system regulates the reproductive axis of turbot according to seasonal changes, especially photoperiod, have not been clearly characterized. In the current study, the cDNA sequences of *kiss2*/*kissr2*, along with *kiss1*/*kissr3* which was thought to be lost in flatfish species, were cloned and functionally characterized. The *kiss1*, *kiss2*, and *kissr3* transcripts were highly detected in the brain and gonad, while *kissr2* mRNA was only abundantly expressed in the brain. Moreover, *kiss*/*kissr* mRNAs were further examined in various brain areas of both sexes. The *kiss1*, *kissr2*, *kissr3* mRNAs were highly expressed in the mesencephalon, while a substantial degree of *kiss2* transcripts were observed in the hypothalamus. During annual reproductive cycle, both *kiss* and *kissr* transcript levels declined significantly from the immature to mature stages and increased at the degeneration stage in the brains of both sexes, especially in the mesencephalon and hypothalamus. The ovarian *kiss1*, *kiss2*, and *kissr2* mRNA levels were highest at the vitellogenic stage (mature stage), while expression of *kissr3* was highest at the immature stage. The testicular *kiss* and *kissr* transcripts were highest in the immature and degeneration stages, and lowest at the mature stage. In addition, intraperitoneal injection of Kiss1-10 and Kiss2-10 significantly stimulated mRNA levels of pituitary *lhβ*, *fhsβ*, and *gthα*. In summary, two Kiss/Kissr systems were firstly proven in a flatfish species of turbot, and it has a positive involvement in controlling the reproduction of the Kiss/Kissr system in turbot. The results will provide preliminary information regarding how the Kiss/Kissr system controls seasonal reproduction in turbot broodstock.

## Introduction

In vertebrates, seasonal reproduction, as the most important biological rhythm, is classically controlled by the hypothalamus-pituitary-gonadal (HPG) axis. Hormones secreted from the HPG axis are regulated by seasonally changing patterns, such as photoperiod, which is an environmental signal that can trigger organism to sexually mature. The discovery of the kisspeptin/GPR54 signaling system has greatly improved our understanding of reproductive endocrinology (1–3). There is an abundance of evidence from mammals demonstrating that the kisspeptin/GPR54 system has a critical role in reproduction, especially in mediating the responses to environmental cues (4, 5). Previous studies in mammals have demonstrated that kisspeptin/GPR54 signaling, as a regulator of the HPG axis, regulates the secretion of gonadotropin-releasing hormone (GnRH) (2, 6). The influence of kisspeptin/GPR54 signaling on reproductive endocrine function has attracted increasing attention from reproductive physiologists.

The kisspeptin/GPR54 system is composed of the ligand, kisspeptin (Kiss), and its receptor, G protein-coupled receptor 54 (GPR54), now renamed kisspeptin receptor (Kissr) (7). The first kisspeptin transcript was isolated from human malignant melanoma cells and its cDNA was designated as Kiss1 (1). Subsequently, the Kiss1 cognate receptor gene (*kissr1*) was characterized in rats (8). Further studies on hypogonadism showed that mutation of *kissr1* with the idiopathic hypothalamic hypogonadism (IHH) syndrome could cause sterile in mice (6, 9). In mammals, the role of the Kiss/Kissr system in the reproductive system was investigated, and has led to significant breakthroughs. It has been demonstrated that the Kiss/Kissr system controls the HPG axis by directly influencing GnRH neurons and regulating the secretion of the GnRH, resulting in the secretion of luteinizing hormone (LH) and follicle-stimulating hormone (FSH) (10, 11).

Following the investigations in mammals, the Kiss/Kissr system has also been characterized in fish species. Parhar and coworkers first reported that another kisspeptin receptor *kissr2* was co-expressed in GnRH neurons in Nile tilapia (*Oreochromis niloticus*), providing valuable insights into the kisspeptin system during normal sexual and reproductive development (12). Subsequently, up to two *kiss* genes (*kiss1*, *kiss2*) and three *kissr* genes (*kissr1*, *kissr2*, *kissr3*) have been identified in various teleosts, including zebrafish (*Danio rerio*) (13–15), medaka (*Oryzias latipes*) (16, 17), goldfish (*Carassius auratus*) (18, 19), European sea bass (*Dicentrarchus labrax*) (20, 21), orange-spotted grouper (*Epinephelus coioides*) (22), chub mackerel (*Scomber japonicas*) (23, 24), yellowtail kingfish (*Seriola lalandi*) (25, 26), European eel (*Anguilla anguilla*) (27, 28), pejerrey (*Odontesthes bonariensis*) (29, 30), lined seahorse (*Hippocampus erectus*) (31), yellowtail clownfish (*Amphiprion clarkia*) (32) as well as four flatfish species, Senegalese sole (*Solea senegalensis*) (33), Atlantic halibut (*Hippoglossus hippoglossus*) (34), Japanese flounder (*Paralichthys olivaceus*) (35), and half-smooth tongue sole (*Cynoglossus semilaevis*) (36, 37).

Kisspeptin has been proven as a key activator of the reproductive axis during the seasonal gonadal cycle in some fish species. For example, changes in expression of *kiss*/*kissr* mRNAs in golden mahseer (*Tor putitora*) and rohu (*Labeo rohita*) indicated that they have a role in gonadal development and annual reproductive cycle (38, 39). Increased transcription levels of the Kiss/Kissr system showed that it is related to the differentiation of the HPG axis during male development in pejerrey (30). Furthermore, kisspeptin neurons in the mediobasal hypothalamus indicated it is a site that is sensitive to feedback from the action of sex steroids in European sea bass (40). The administration of exogenous kisspeptin peptides has been shown to promote plasma levels of FSH and LH in several fish species (18, 31, 41, 42). However, Kiss knockout in zebrafish and medaka, the puberty onset and sexual differentiation are not affected (43–45). Kiss2 administration in recrudescent fish do not change the blood LH levels (43). It might be species-specific in the control of reproduction in teleosts. Of note, kisspeptin expression revealed specific patterns that coincided with seasonal environmental changes, more specifically photoperiod, and induced sexual development. Intracerebroventricular infusion of Kiss1 could override the inhibitory photoperiod and reactivate sexual activity in hamsters which are sexually active in long photoperiod and quiescent in short photoperiod (43, 46). Kiss1 in mice showed circadian patterns that peaked coincident with LH (46). Similarly, Kiss controlled of cyclic reproductive activity in teleost. Oocyte maturation, responding to day length, relied on the enhanced transcription of *kiss* and *gnrh3* in the brain of zebrafish (47). The rhythms of *kiss* and *gnrh/fshβ/lhβ* mRNA expression further suggested that the natural photoperiod is involved in activating the reproductive axis during maturation in Atlantic cod (*Gadus morhua*), European sea bass and grass puffer (*Takifugu niphobles*) (48–50).

Turbot (*Scophthalmus maximus*) is classified into the Pleuronectiformes, and is one of the most commercially important fish species cultured in Europe and North China. During turbot aquaculture, photoperiod manipulation is used to induce egg and sperm production on a year-round basis (51–53). Previous studies have shown that exposure to continuous light reduced the proportion of females that mature and egg production by as much as 90% (54). Similarly, the administration of a long-term photoperiod around the spring equinox significantly decreased and delayed maturation in adult turbot (51, 55). Based on the previous studies, we hypothesized that there has a Kiss/Kissr system in turbot involved in the regulation of the entire reproductive axis in response to seasonal changes. Therefore, in this study, we aimed to identify the Kiss/Kissr system in turbot and investigate the expression profiles of kisspeptin genes in the brain and gonad of turbot within an annual reproductive axis under culture conditions exposed to artificial light. Moreover, the physiological role of Kiss1 and Kiss2 in regulating pituitary hormone gene expression was also examined. These data provide crucial information for controlling seasonal reproduction and breeding of turbot broodstock.

## Materials and Methods

### Fish and Samples

The turbot individuals used in this study were obtained from a fish farm in Shandong Province, China. All of the broodstock were over 2 years old and had previously undergone gonadal maturation. The fish were cultured under a simulated natural photoperiod (12L:12D) and ambient temperature of 17 ± 0.8°C before the reproductive season. During the breeding season, from November 2018 to April 2019, the lighting regime progressed through three stages: an inhibitory photoperiod (8L:16D) for about 1.5 months, a natural photoperiod (12L:12D) for about 0.5 months, and a prolonged photoperiod (16L:8D) for about 4 months. The fish were fed with enhanced nutrition (small yellow croaker *Larimichthys polyactis* and Pacific sand lance *Ammodytes personatus*). Three males and three females were collected for sampling every month throughout the reproductive season. Before sampling, turbots were anesthetized with a 0.05% MS222 (Sigma-Aldrich). The gonad from each turbot was divided into two parts, one part was fixed in Bouin’s fluid for histological analysis and the other was frozen in liquid nitrogen and then stored at -80°C. Besides gonads, brain and pituitary gland were also collected for qPCR analysis. In addition, various tissues, including brain, eye, skin, muscle, gill, liver, heart, spleen, kidney, stomach, intestine, liver, and gonad from immature male turbot were collected and stored at -80°C to examine the tissue distribution of the Kiss/Kissr system.

### Histological Analysis

The fixed gonadal tissue samples were dehydrated through a graded series of alcohol concentrations and embedded in paraffin. Serial sections of 5 μm were sliced using a rotary microtome (Leica, Germany) and treated with HE (Haematoxylin and Eosin) stain. Stained sections were examined using a microscope Axio Scope A1 (Leica, Germany). The developmental stages of gonads were confirmed based on ovarian and testicular histology. All of the collected turbots were classified into II, III, IV, V, and VI stages according to the dominant gamete type and characteristics (56, 57).

### Molecular Cloning

Total RNA was extracted from the brains of immature turbot using the RNA Isolation Kit (Catalog No. 220011) (Fastagen Biotech, China). Quality and concentration of RNA were measured using 1.0% agarose gels electrophoresis and assessed by spectrophotometry (ND-2000, Nanodrop, USA). The first-strand cDNA was synthesized using a TransScript First-Strand cDNA synthesis kit (AT301-02) (Transgen, China) following the manufacturer’s instructions. Subsequently, cDNA fragments of turbot *kiss1*, *kiss2*, *kissr2*, and *kissr3* were amplified with primers (**Table 1**). Finally, 5’-RACE and 3’-RACE were performed for full-length cDNAs using the SMARTer RACE cDNA Amplification Kit (Catalog No.634923) (Clontech, USA) with gene-specific primers (GSPs) (**Table 1**). The diluted first-round PCR products were used as templates for nested PCR with corresponding primers of GSP-nest (**Table 1**). All PCR programs were performed using a PTC-100 thermal cycle (Bio-Rad, USA). PCR was performed in a 25 μl reaction volume containing 1 μl of diluted cDNA, 1 μl of each primer (10 μM), 12.5 μl of Premix buffer (with dNTPs), and 9.5 μl ddH_2_O, with amplification procedure of denaturation at 94°C for 5minutes; 35 cycles of amplification at 94°C for 30 seconds, annealing with specific temperature of each primer (**Table 1**) for 45 seconds, elongation with 72°C for 30 seconds, an additional elongation at 72°C for 10 minutes.

**Table 1:** List of primers used for molecular cloning of kiss and kissr cDNAs.

Primer name	Nucleotide sequence (5’-3’)	Temperature of annealing (°C)	Purpose
kiss1-F	ACCTGCTGAYARRGTCCAKTCAG	58	Fragment PCR (138bp)
kiss1-R	TTTCCRTAACGKAGACCAAAGG		
kiss2-F	GCTCTGGTTGTTGTGTGCG	59	Fragment PCR (310bp)
kiss2-F	TCCTGGCTCTTTTAACGGCT		
Kissr3-F	CCACACTGTACCCTCTGCCCAGCTG	59	Fragment PCR (626bp)
Kissr3-R	TTGACGGAGGAGTTGGAGTA		
kissr2-F	GGMAACTCWCTGGTSATTTATGT	58	Fragment PCR (833bp)
kissr2-R	AYTTGGCGTAGGACATGCAGTT		
5’- kiss1-GSP1	TCCATAACGGAGACCAAAGGAGTTGAGGTT	65	5’ RACE-PCR
5’- kiss1-GSP2	GATCCACCATCCTGATCTGGGAAACT	63	5’ RACE-PCR(nested)
3’- kiss1-GSP1	CTGATAAGGTCCATTCAGCTGATGGAAAGT	62	3’ RACE-PCR
3’- kiss1-GSP2	CATACAACCTCAACTCCTTTGGTCTCCG	63	3’ RACE-PCR(nested)
5’- kiss2-GSP1	GCTCTCCTGTAGATGTAGCGTTTCCCGA	63	5’ RACE-PCR
5’- kiss2-GSP2	TCCTCCTCCTGAGCGCGGAGAGGAC	60	5’ RACE-PCR(nested)
3’- kiss2-GSP1	ATGAGGCTTGTGGCTCTGGTTGTTGTG	63	3’ RACE-PCR
3’- kiss2-GSP2	GCTGTGCAACGACCGCAGGAGCAA	60	3’ RACE-PCR(nested)
5’- kissr3 -GSP1	GGGCACACAGCAGACCAGGAACAAGAT	65	5’ RACE-PCR
3’- kissr3 -GSP1	GTCTTACTCCAACTCCTCCGTCAACCC	65	3’ RACE-PCR
5’- kissr2 -GSP1	CAGAAGGAGATAGTCAGGACGGGCAG	65	5’ RACE-PCR
5’- kissr2 -GSP2	ATTTCAGAGGGTAGACGGTGACATAG	66	5’ RACE-PCR(nested)
3’- kissr2 -GSP1	CCAAACACAGGCAGATGAGGACGGCG	66	3’ RACE-PCR
3’- kissr2 -GSP2	ATAAGCATCAGAAGCAAAGTCTCCAA	65	3’ RACE-PCR(nested)

### Phylogenetic Analysis

Homology searches of the deduced turbot Kiss1, Kiss2, Kissr2, and Kissr3 sequences were performed using the National Center for Biotechnology Information website (http://www.ncbi.nlm.nih.gov/). The putative signal peptides were predicted by SignalP4.1. The putative transmembrane domain was predicted using the TMHMM server V2.0. The percentages of similarity and identity were calculated using LALIGN (http://www.ch.embnet.org/software/LALIGN-form.html). Multiple alignments of predicted amino acid sequences were conducted using BioEdit with the ClustalW alignment tool. A phylogenetic tree was constructed in the Mega7 software by the neighbor-joining method using bootstrapping with over 1,000 iterations.

### Synteny Analyses of kiss1 and kiss2 Genes

The syntenic analysis of kiss1 and kiss2 genes among turbot, zebrafish, medaka and tilapia was conducted basing on the comparison the neighboring genes. Briefly, the protein sequences of neighbor genes of Kiss were predicted from the turbot chromosome sequences by FGENESH program. The identified neighboring protein sequences were annotated against NCBI by BLASTP. The conserved syntenic pattern of Kiss genes in other species were determined in Ensembl database (http://asia.ensembl.org/index.html) and Genomicus (https://www.genomicus.bio.ens.psl.eu/genomicus-83.01/cgi-bin/search.pl).

### Administration of Kiss1 and Kiss2 to Turbot

According to the sequence analysis, turbot Kiss1-10 (YNLNSFGLRY-NH2) and Kiss2-10 (FNFNPFGLRF-NH2) peptides were synthetized by ChinaPeptides Co., Ltd. (Shanghai, China) with purities of 98% and 97%, respectively, as determined by HPLC. For *in vivo* experiments, juvenile turbot (body weight (BW) = 9.575 ± 0.47 g) were used, at a rearing temperature of 16 ± 0.5°C with dissolved oxygen > 7.0 mg/L and a 12L:12D photoperiod.

The doses of Kiss peptides referred the administration experiments in some fish species, including goldfish and cinnamon clownfish (18, 31, 42). In these articles, it demonstrated that intraperitoneal injection of 0.01-1ug/g in 6h could stimulate expression of *lhβ* and *fshβ*. Therefore, doses of 100ng/g BW and 1000 ng/g BW with intraperitoneal injection were chosen. The synthetized peptides were dissolved in phosphate-buffered saline (PBS) and injected intraperitoneally into turbot anesthetized with 0.05% MS222. PBS alone acted as the negative control. At 3 and 6 h post-injection, sample fish were collected (n = 6) and their pituitary glands quickly dissected, frozen in liquid nitrogen, and stored at -80°C for qPCR analysis.

### Quantitative Real-Time PCR (qPCR)

Quantitative real-time PCR was performed using the CFX96 Real-Time PCR Detection System (Bio-Rad, USA) with a SYBR Premix Ex Taq Kit (Takara, Japan) using the standard curve method with *β-actin* as the reference gene. Specific primers used for each target gene were designed by the Primer Premier 5.0 software (**Table 2**). The 20 μl reaction system contained 10 μl SYBR Premix Ex Taq, 0.2 μl forward primer (10 μM), 0.2 μl reverse primer (10 μM), 7.6 μl ddH_2_O, and 2 μl cDNA template. The PCR procedure was programmed according to the manufacturer’s protocol: 30 s at 95°C, 5 s at 95°C, and 30 s at 60°C for 40 cycles. A dissociation curve was performed at the end of each program to determine the amplification specificity. The relative gene expression levels were analyzed by the 2^- ΔΔCT^ method.

**Table 2:** List of primers used for qPCR expressions analysis of kiss and kissr mRNAs.

Primer name	Nucleotide sequence (5’-3’)	Products (bp)	E	R^2^
sm-kiss1-F	GCCTTGAGAGATTTAACCGATGC	143bp	0.93	0.991
sm-kiss1-R	ATAGAGTGAGGGAGGACCAAGTT			
sm-kiss2-F	GTGGATGAACGTTAACAGGCAC	142bp	0.97	0.996
sm-kiss2-R	GTCGAGTCATATCCTGGCAGAG			
sm-kissr3-F	GGTTTACGCCTTCATGGGCA	124bp	0.98	0.996
sm-kissr3-R	GTCCTTCCCTTCCTCCGCTT			
sm-kissr2-F	CAGCGTCTGCATTTGGATCG	164bp	1.03	0.998
sm-kissr2-R	AGGCGGCGATAAACTGGTAG			
sm-fshβ-F	TCGGCTGCAAACTGGC	185bp	1.02	0.997
sm-fshβ-R	ATCCGTTAATGTGCTTCG			
sm-lhβ-F	CAAGGAACCCTCCATCATCTTT	96bp	0.99	0.997
sm-lhβ-R	AGCTGCACCGTCCTGTAGTG			
sm-gthα-F	ACCCGACACCACTCAAGACA	148bp	0.97	0.998
sm-gthα-R	CTTTTATGCCGACACCCACA			
sm-β-actin-F	GCTGTGCTGTCCCTGTATGCC	187bp	0.92	0.997
sm-β-actin-R	AGGAGTAGCCACGCTCTGTCA			

E, reaction efficiencies; R2, Pearson’s coefficients of determination.

### Statistical Analysis

Statistical analysis was carried out using SPSS version 21.0. All results are presented as means ± SEM. Gene expression during gonadal stages were analyzed using one-way analysis of variance (ANOVA) followed by Duncan’s multiple range tests. Differences were considered to be significant at *P* < 0.05. All assays were carried out independently in triplicate.

## Results

### Molecular Cloning of *kiss*/*kissr* in Turbot

The full-length cDNA of turbot *kiss1* (GenBank accession no. MW057929) was 580 bp in size, with an open reading frame (ORF) of 312 bp encoding a preprohormone of 104 amino acids. The full-length cDNA of turbot *kiss2* (GenBank accession no. MW057930) was 622 bp, with an ORF of 369 bp encoding a precusor of 123 amino acids. Amino acid sequence analysis demonstrated that an obvious Kiss1 domain-YNLNSFGLRY (Kiss1-10) and Kiss2 domain-FNFNPFGLRF (Kiss2-10) were involved in turbot kisspeptin system (**Supplementary Figure 1**). However, the Kiss1-10 differed from Kiss2-10 by 4 amino acids in turbot. The sequence alignments of the deduced amino acid sequences of turbot Kiss1 and Kiss2 with other vertebrate species are presented in **Supplementary Figure 2**.

Notably, although the Kiss-10 regions are highly conserved across vertebrates, there were still 2 positions in Kiss1-10 and 3 positions in Kiss2-10 with different amino acids. For Kiss1-10, at the 3^rd^ position from the N-terminus, most teleosts exhibit a Leucine and some exhibit phenylalanine, while mammals and amphibians exhibit a Tryptophan. For turbot Kiss2-10, the 3^rd^ position from the N-terminus was identical to medaka, zebrafish, longtooth grouper (*Epinephelus bruneus)*, rare minnow (*Gobiocypris rarus)*, and Senegalese sole. However, turbot had Phenylalanine and Glycine at positions 6 and 7 from the N-terminus, which differed from the Leucine and Threonine in Japanese flounder.

**Figure 1:**
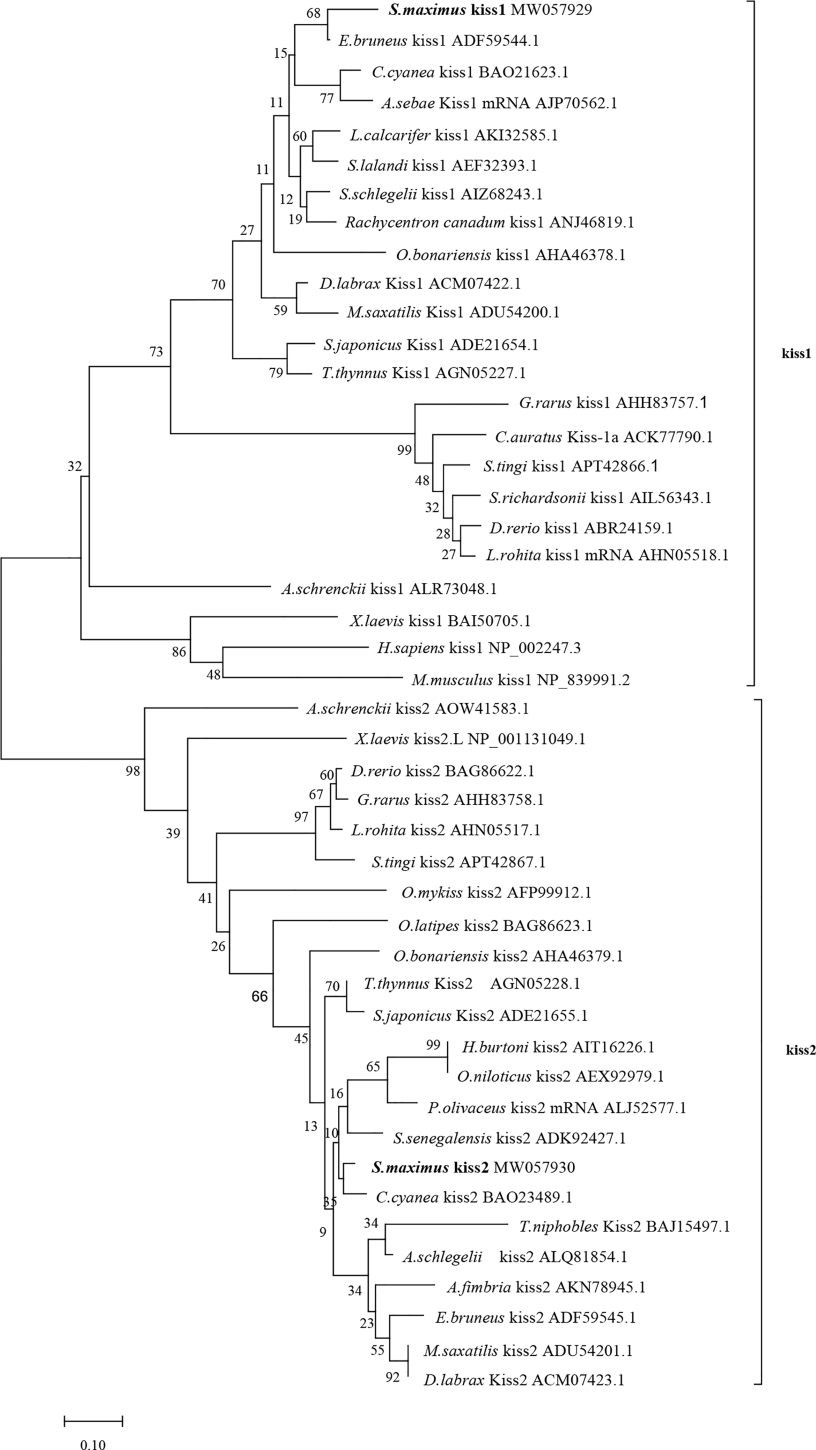
Phylogenetic tree of deduced amino acid sequences of Kiss1 and Kiss2 from turbot and other vertebrates was constructed by Mega7 with neighbor-joining method. The bootstrap values at nodes indicate bootstrap percentage value for 1000 replicates (>80%). Relative branch lengths indicate the evolutionary rates of the lineages. The GenBank accession numbers of the sequences were presented after the species name.

**Figure 2:**
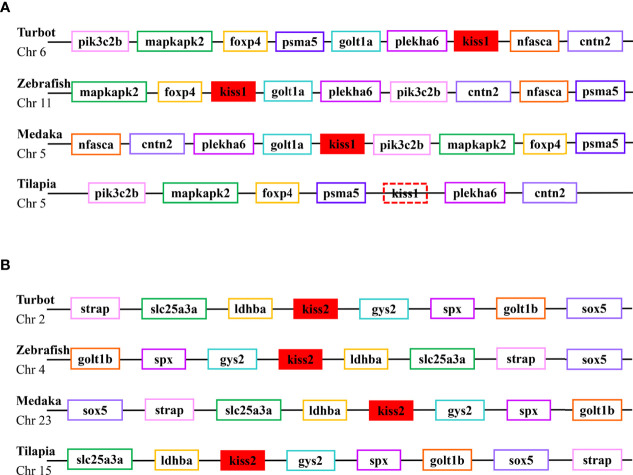
Conserved synteny for the genomic region of *kiss1* **(A)** and *kiss2* **(B)** genes.

Phylogenetic analysis revealed that the Kiss1 and Kiss2 sequences clustered in two separate clades (**Figure 1**). The synteny analysis of kiss showed that the *kiss1* gene was usually positioned in the genomic regions, including *plekha6*, *cntn2*, *psma5*, *foxp4*, *mapkapk2*, and *pik3c2b*, and *kiss2* gene was usually positioned including *gys2*, *spx*, *golt1b*, *ldhba*, *slc25a3a*, and *strap* (**Figure 2**).

**Figure 3:**
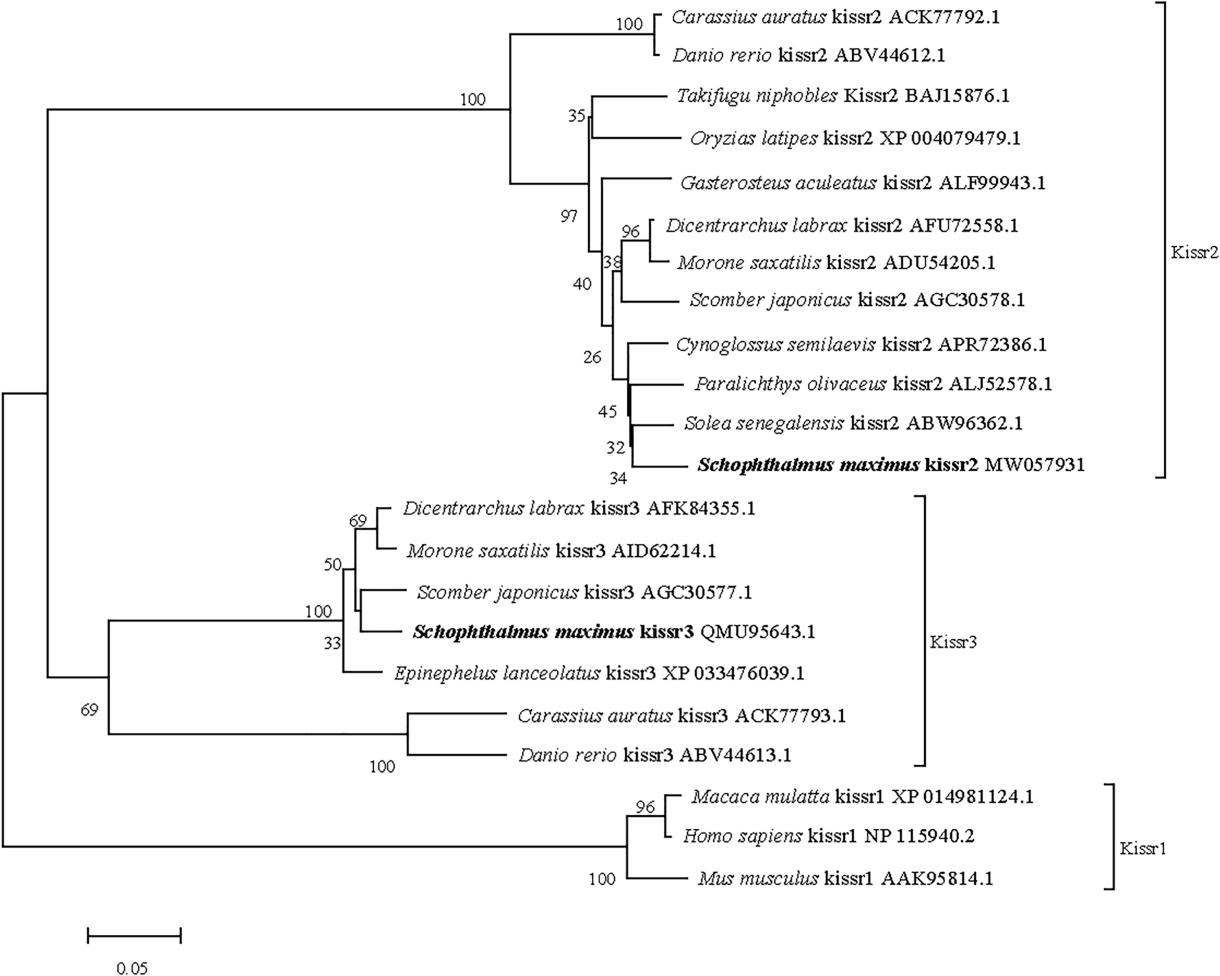
Phylogenetic tree of deduced amino acid sequences of Kissr3 and Kissr2 from turbot and other vertebrates was constructed by Mega7 with neighbor-joining method. The bootstrap values at nodes indicate bootstrap percentage value for 1000 replicates (>80%). Relative branch lengths indicate the evolutionary rates of the lineages. The GenBank accession numbers of the sequences were presented after the species name.

The full-length cDNA of turbot *kissr3* (GenBank accession no. MT319113) was 2329 bp, with an ORF of 1092 bp encoding a peptide with 363 amino acids. The full-length cDNA of turbot *kissr2* (GenBank accession no. MW057931) was 1578 bp, with an ORF of 1128 bp encoding a peptide with 375 amino acids. The deduced amino acids of Kissr3 and Kissr2 all contained a seven transmembrane domain (**Supplementary Figure 3**). The sequence alignment of the deduced amino acid sequences of turbot Kissr2 and Kissr3 to those of other vertebrate species is presented in **Supplementary Figure 4**. In the phylogenetic analysis, the two turbot Kissr clustered into the Kissr2 and Kissr3 clades, respectively (**Figure 3**).

### Tissue Distribution of *Kiss* and *Kissr* Transcripts

**Figure 4:**
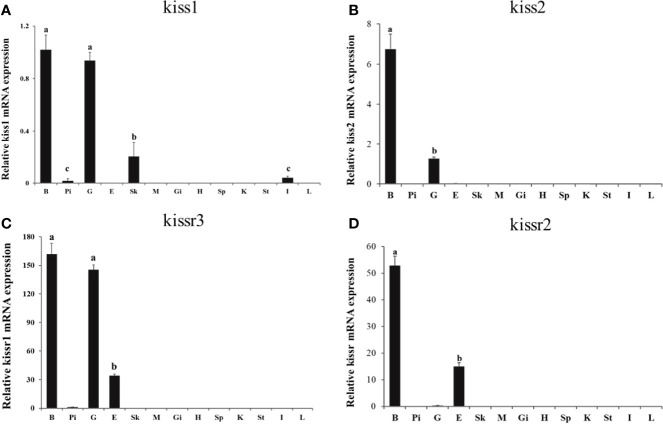
Tissue distribution of *kiss/kissr* genes in turbot. **(A)** kiss1, **(B)** kiss2, **(C)** kissr3, **(D)** kissr2. The relative abundance of kiss/kissr genes were expressed as mean ± SEM (n=3). Tissue abbreviations: B, brain; Pi, pituitary; G, gonad; E, eye; Sk, skin; M, muscle; Gi, gill; H, heart; Sp, spleen; K, kidney; St, stomach; I, intestine; L, liver. Different letters above bars represent statistical significance (p < 0.05) between two different tissues.

Expression patterns of turbot *kiss* (*kiss1*, *kiss2*) and *kissr* (*kissr2*, *kissr3*) genes in various tissues and different brain regions were detected by qPCR. Firstly, expression of tissues of brain, pituitary, gonad, eye, skin, muscle, gill, heart, spleen, kidney, stomach, intestine, and liver from adult male were shown in **Figure 4**. The *kiss1* mRNA was highly expressed in the brain and gonad, but had low expression in the skin, pituitary, and intestine. The *kiss2* mRNA was particularly highly expressed in brain and gonad, and barely expressed in other tissues. The *kissr3* mRNA was significantly expressed in brain, gonad, and eye. While the *kissr2* mRNA was abundantly expressed in brain and eye, but barely in gonad.

**Figure 5:**
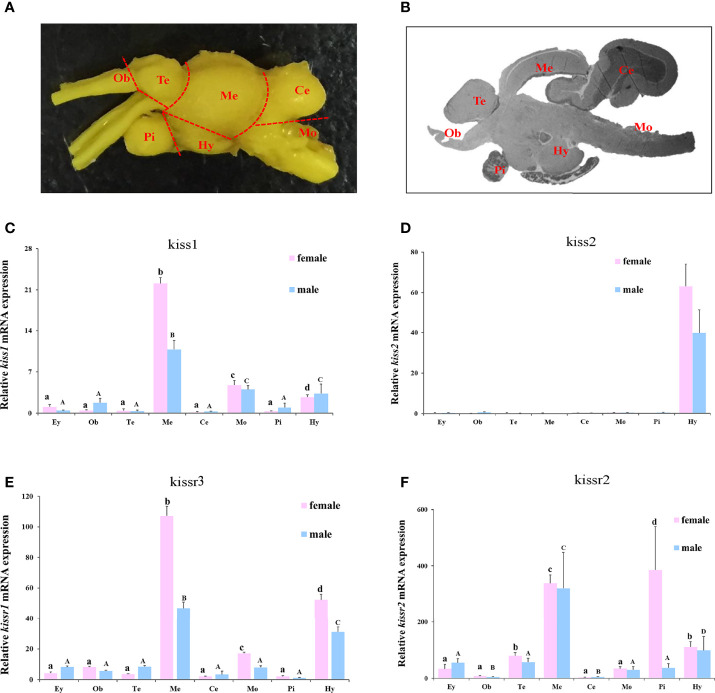
The turbot brain areas and expression of *kiss*/*kissr* genes in different brain areas and eyes. **(A)** morphology of turbot brain areas. **(B)** histology of turbot brain areas. **(C)** *kiss1*. **(D)** *kiss2*. **(E)** *kissr3*. **(F)** *kissr2*. The relative abundance of *kiss*/*kissr* genes were expressed as mean ± SEM (n = 3). Ey, eye; Ob, olfactory bulbs; Te, telencephalon; Me, mesencephalon; Ce, cerebellum; Hy, hypothalamus; Pi, pituitary gland; Mo, medulla oblongata. The different uppercase letters in the figure represent statistical significance (*p < 0.05*) in male. The different lowercase letters in the figure represent statistical significance (*p < 0.05*) in female.

The expression patterns of turbot *kiss/kissr* mRNAs in central brain areas, including olfactory bulbs (Ob), telencephalon (Te) with preoptic area (POA), mesencephalon (Me) with thalamus, hypothalamus (Hy) with saccus vasculosus, cerebellum (Ce), medulla oblongata (Mo), and pituitary gland (Pi) were also analyzed (**Figure 5**). The expression in the eye was also investigated alongside the central brain areas. The *kiss/kissr* mRNAs were expressed in all central brain areas and eyes. The *kiss1* mRNA expression was high in Me, but low in Mo and Hy. However, the *kiss2* mRNA was highly detected in the Hy. The expressions of *kissr3* and *kissr2* mRNA were high in the Me and Hy. In addition, the expression patterns of *kiss/kissr* mRNAs were similar in the female and male turbot brains, however, expression levels were higher in the central brain areas of females than males.

### Gonadal Morphology and Histology Assessment of Reproductive Stages

**Figure 6:**
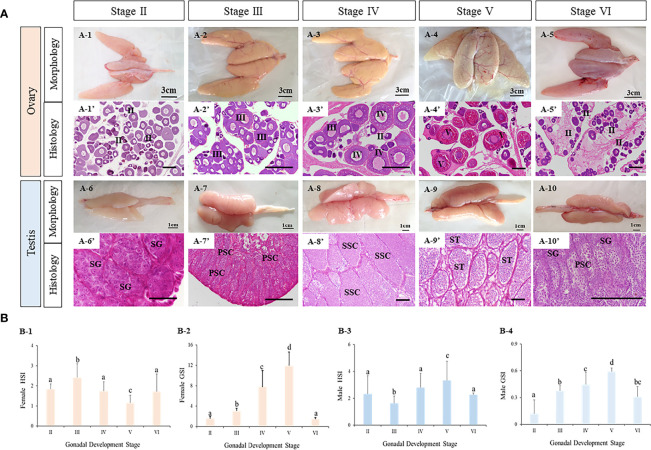
The characteristics of tourbot testes and ovaries during reproductive cycle. **(A)** morphology and histology of turbot testes and ovaries. A1-5, morphology of turbot ovaries. A1’-5’, histology of turbot ovaries. A6-10, morphology of turbot testes. A6’-10’, histology of turbot testes. **(B)** GSI and HSI of turbot testes and ovaries. B1-2, GSI and HSI of female turbot. B3-4, GSI and HSI of male turbot. **(A)** PSC, primary spermatocytes; SG, spermatogonia; SSC, secondary spermatocytes; ST, spermatids; SZ, spermatozoa; **(B)** I, previtellogenic oocytes. II, primary vitellogenic oocytes; III-IV, large growth of vitellogenic oocytes. Scale: A1-5, 3 cm; A6-10, 1 cm; A1’-10’, 50 μm. Different letters above bars represent statistical significance (*p < 0.05*) at different reproductive stages in **(B)**.

The morphology and histology of ovaries and testes during breeding season of turbot were examined. Five ovarian and five testicular developmental stages were identified from stage II to VI (**Figure 6**). First, the five ovarian stages are shown in **Figure 6A1–5**. At stage II, the ovary was present with light fleshy red color and the blood vessels on the ovarian membrane were not obvious. The germinal epithelium of the ovary was filled with oocytes (primary growth oocytes and perinucleolar oocytes) in stage II. At stage III, the appearance of the ovary was flesh-colored, and eggs could be seen in the ovary. The histological results showed the oocytes of stage III had developed into the previtellogenic stage and vitellogenesis had begun. At stage IV, the ovary exhibited significant growth and the oocytes were characterized as late vitellogenic. At stage V, matured oocytes were obvious in the ovary and the proportion of oocytes in the post vitellogenic stage increased. Stage VI was characterized by degenerated ovaries and there were only primary growth oocytes left.

Similarly, the appearance and major cell types in the five developmental stages of testis were observed (**Figure 6A6–10**). With development, the testis became bigger and wrinkles and blood vessels became clear. In addition, spermatogenesis was observed. At stage II, the testis contained a large number of spermatogonia. At stage III, spermatogenesis had begun and most germ cells had become primary spermatocytes. At stage IV, secondary spermatocytes appeared. At stage V, the seminiferous tubules were filled with spermatozoa with tails, indicating that spermatogenesis was nearly complete. With the release of spermatozoa, the testis were classified as stage VI.

Meanwhile, the gonadosomatic index (GSI, gonad weight/body weight) and hepatosomatic index (HSI, liver mass/body weight) were calculated (**Figure 6B1–4**). In both male and female turbot, the GSI of ovaries and testes all increased from stage II to V, reaching their peak values at stage V, before decreasing at stage VI. The HSI of ovaries at stage V was lower than all other stages. While in testes, the HSI was highest at stage V.

### Expression Patterns of *Kiss* and *Kissr* Transcripts in the Brain During Reproductive Stages

**Figure 7:**
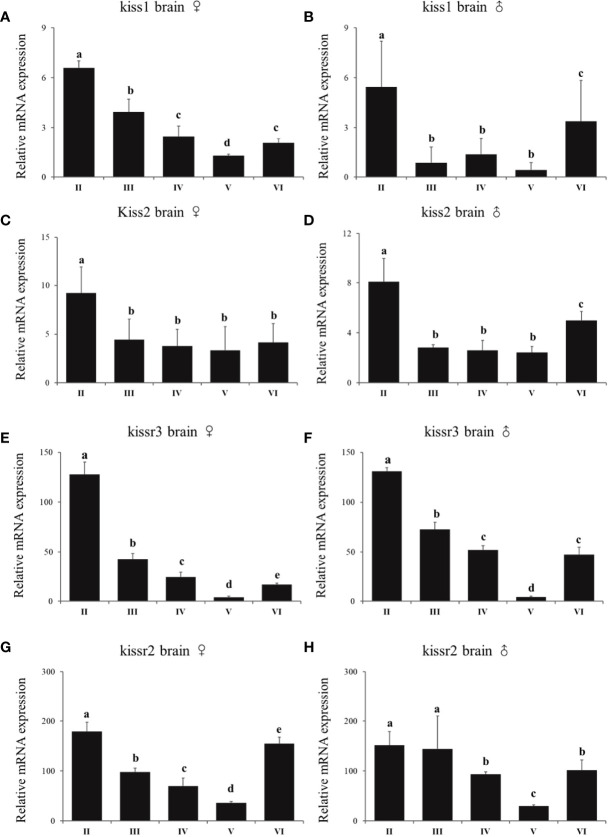
Expression profiles of *kiss/kissr* genes throughout the reproductive cycle in the brains of female **(A, C, E, G)** and male **(B, D, F, H)**. Error bars are presented as the mean±SEM. Different letters above bars represent statistical significance (p < 0.05) between two reproductive stages.

In females, *kiss1* mRNA expression was very high during stage II, then gradually decreased from stage II to V, followed by a significant increase at stage VI (**Figure 7A**). The *kiss2* expression level declined significantly from stage II to III, and did not change significantly from stage III to VI (**Figure 7C**). Meanwhile, the *kissr3* and *kissr2* mRNA expression profiles showed a similar pattern to *kiss1* mRNA expression (**Figures 7E, G**). In males, the expression patterns of *kiss1* and *kiss2* mRNA were similar during the reproductive stages (**Figures 7B, D**). The highest expression levels appeared at stages II and VI. Their expression levels did not change significantly from stage III to V, but were significantly lower than stages II and VI. In addition, the expression patterns of *kissr3* and *kissr2* mRNA in males were similar to those in female turbot (**Figures 7F, H**).

**Figure 8:**
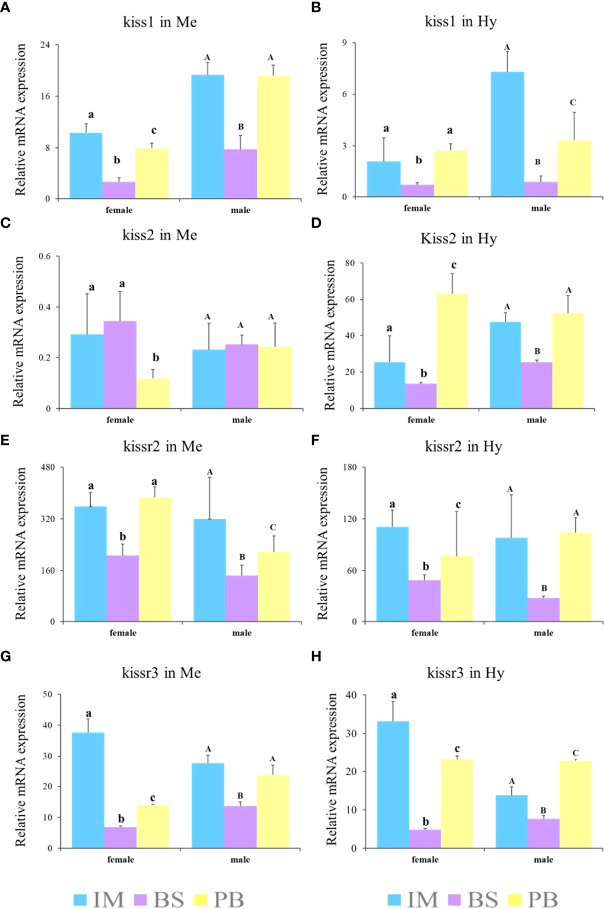
Expression profiles of *kiss/kissr* genes in Me **(A, C, E, G)** and Hy **(B, D, F, H)** at immature stage (IM), the breeding season (BS) and post-breeding season (PB). Error bars are presented as the mean±SEM. The different lowercase letters in the figure represent statistical significance (p < 0.05) between two stages in female. The different uppercase letters in the figure represent statistical significance (p < 0.05) between two stages in male.

Furthermore, to explore expression patterns in different brain regions, the expression levels of *kiss* and *kissr* transcripts in the Me and Hy of both sexes during three gonadal development stages, immature stage (II), mature stage (IV-V), and degeneration stage (VI), were analyzed (**Figure 8**). The results showed that the expression levels of *kiss* and *kissr* transcripts declined significantly from the immature to mature stages, and increased at the degeneration stage. However, *kiss2* expression in the Me of females was lowest at the degeneration stage. In males, *kiss2* expression in the Me showed no significant differences among the different gonad developmental stages.

### Expression Patterns of *Kiss* and *Kissr* Transcripts in the Gonad During Reproductive Stages

**Figure 9:**
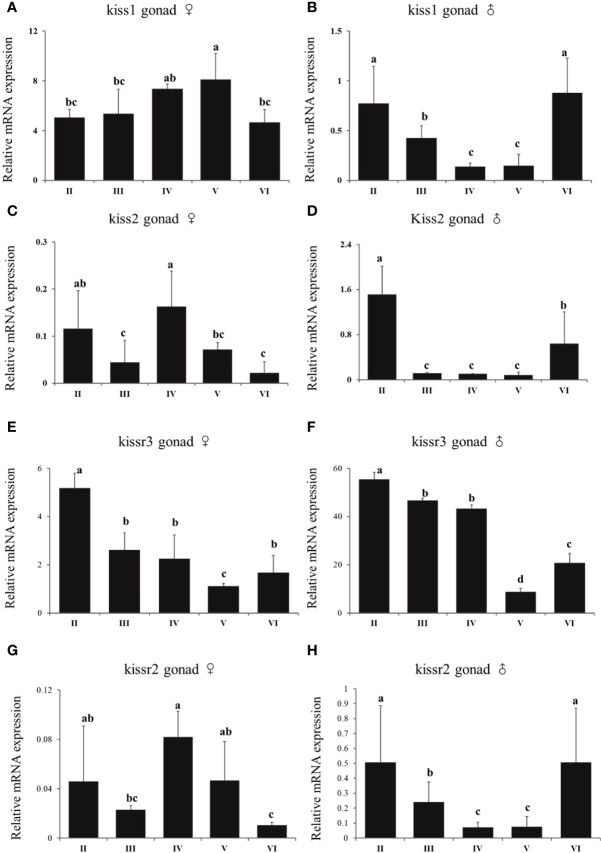
Expression profiles of *kiss/kissr* genes throughout the reproductive cycle in the gonads of female **(A, C, E, G)** and male **(B, D, F, H)**. Error bars are presented as the mean±SEM. Different letters above bars represent statistical significance (p < 0.05) between two reproductive stages.

In females, there was no a distinct trend of expression of kiss/kissr system. There was no difference of expression of *kiss1* in stage V and IV. However, the expression of *kiss1* in stage V was higher than that in stage II, III, and VI (**Figure 9A**). There was high expression of *kiss2* and *kissr2* mRNA at stage IV (**Figures 9C, G**). For *kiss2*, the lowest expression happened at stages III and VI. The lowest expression of *kissr2* happened at stage VI. The highest *kissr3* mRNA expression level appeared at the immature stage (II) (**Figure 9E**). In males, the highest *kiss* and *kissr* mRNA expression levels appeared at stages II and VI, and lowest at the mature stage (V) (**Figures 9B, D, F, H**).

### 3.6 Effects of Administration of Kiss1-10 and Kiss2-10 on the Pituitary of Turbot

**Figure 10:**
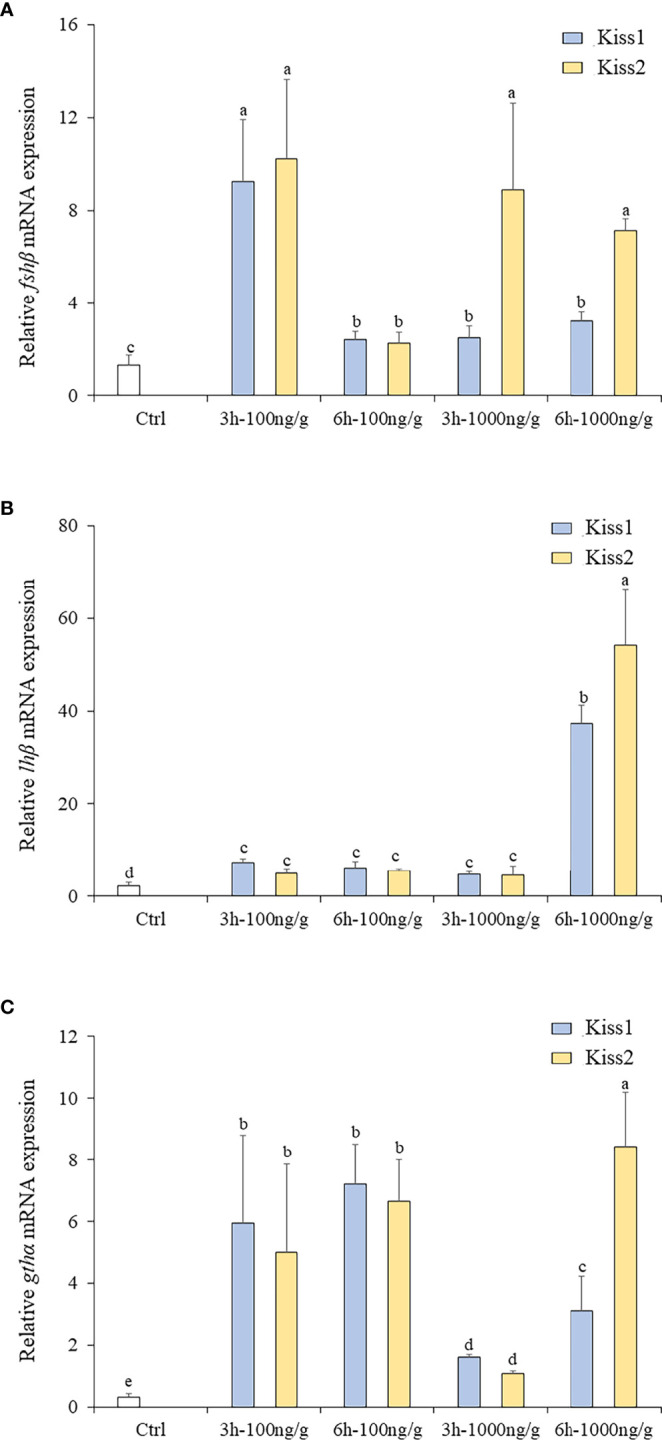
Expression profiles of *fhsβ* **(A)**, *lhβ* **(B)** and *gthα* **(C)** in the pituitary of turbot by administration of Kiss1-10 and Kiss2-10 at 3 and 6 h post-injection. Error bars are presented as the mean ± SEM. Different letters above bars represent statistical significance (*p < 0.05*).

The changes of *fshβ*, *lhβ*, and *gthα* transcription levels were analyzed to investigate the physiological effects of Kiss1-10 and Kiss2-10 on pituitary function. The *fshβ* (**Figure 10A**), *lhβ* (**Figure 10B**), and *gthα* (**Figure 10C**) transcription levels increased significantly at 3 h and 6 h post-injection of 100 ng/g and1000 ng/g Kiss1-10 and Kiss2-10. The transcription levels of *fshβ* peaked at 3 h under 100 ng/g of Kiss1-10 and Kiss2-10, and 1000 ng/g of Kiss2-10. The *lhβ* transcription reached the highest level at 6 h under 1000 ng/g of Kiss1-10 and Kiss2-10. *Gthα* transcription increased significantly from 3 h to 6 h under the administration of both doses. It was note that, after 3h injection, the *fshβ* and *Gthα* mRNA had a lower expression with 1000ng/g than that with 100ng/g.

## Discussion

In the present study, we cloned and characterized the Kiss/Kissr system (*kiss1*, *kiss2*, *kissr2*, *and kissr3*) in turbot, and provided preliminary information on its roles throughout the seasonal reproductive period. To date, numerous studies have reported on the Kiss/Kissr system in vertebrates. In most mammals, there is only one kisspeptin gene, *kiss1*, except in the platypus which contains two forms, *kiss1* and *kiss2* (58). However, two forms of *kiss* genes have been identified in several fish species, such as zebrafish, medaka, goldfish, European seabass, among others (59). However, in almost 20 fish species, only *kiss2* can be found, such as the three-spined stickleback (*Gasterosteus aculeatus*), Nile tilapia (*Oreochromis niloticus*), and orange-spotted grouper (*Epinephelus coioides*) (20, 22, 60, 61).

Until now, four *kiss* and four *kissr* genes have been identified, indicating the Kiss/Kissr system has a high diversity in vertebrates (62). In this study, two *kiss* genes (*kiss1* and *kiss2*) and two *kissr* genes (*kissr3* and *kissr2*) were isolated in turbot. However, previous studies in other Pleuronectiformes only found *kiss2* and *kissr2*, including Japanese flounder (35), half-smooth tongue sole (36, 37), and Senegalese sole (63). Thus, a hypothesis that *kiss1* was probably lost in Pleuronectiformes was put forward. Many researchers have tried to explain the lack of *kiss1* and establish a clearer picture of their molecular diversity in evolutionary terms of whole genome duplication (35, 62). While teleost-specific whole genome duplication (also known as the third round,3R) should have generated additional *Kiss* paralogs, there is a loss of *Kiss1* in some teleosts (62). The reason might be a massive loss of 3R-Kiss paralogs shortly after the 3R event. However, according to synteny analysis and cognate neighboring genes, it is confirmed that turbot possesses *kiss1* gene. It’s worth noting that Pleuronectiformes was split into ‘real flatfish Pleuronectoidei’ (RFP) and ‘flatfish-like Psettodoidei’ (FLP) lineages by genome analyses, and it was confirmed a polyphyletic origin for these two lineages in recent research (64). Meanwhile, there were morphological and genetic difference between the two lineages. Though it is clustered into RFP, the turbot seemed to be closer to FLP which forms one clade with Perciformes species according to the phylogenetic analysis. Therefore, it is supposed that turbot might manage to maintain *kiss1* as an exceptional case within Pleuronectiforms.

Generally, the *kiss* gene encodes a polypeptide precursor, which comprises the mature peptide kisspeptin. Matured kisspeptin peptides encompass a C-terminal 10- decapeptide, namely Kiss-10 (20, 58). Positions 1 and 10 correspond to aromatic amino acids that are fully conserved among vertebrates, and consists of the “Y-Y type” and “F-F type” forms (35). In this study, turbot Kiss1-10 was YNLNSFGLRY, belonging to the “Y-Y type”, and Kiss2-10 was FNFNPFGLRF, belonging to the “F-F type”. Positions 3 and 5 of Kiss1-10 and positions 3, 7, and 8 of Kiss2-10 always exhibited variations in this study. This feature may help in generating specific antibodies against distinct kisspeptins for functional investigations (61).

The receptors of Kiss ligands were GPR54, now known as Kissr, and were first discovered in the rat (8). Previous studies showed that Kissr have been highly conserved throughout evolutionary history. The amino acid sequence comprised seven transmembrane helices (TM1 to TM7) and has a high shared identity among different vertebrate species (58). To date, four Kissr paralogous have been described. In teleosts, all species investigated so far have possessed the *kissr2* gene, indicating the existence of one ancestral Kiss receptor (7). In this paper, phylogenetic analyses have revealed that turbot has two *kissr* paralogous, *kissr2* and *kissr3*, as in some fish species, including zebrafish, medaka, and chub mackerel (14, 58, 65). In addition, three different *kissr* were found in the European eel (*Anguilla anguilla*) (28), which suggested that teleost species also possess a diversity of *kissr* genes.

Tissue expression profiles of *kiss* and *kissr* genes have been reported in many fish species (9, 35, 59). Generally, the highest expression of *kiss*/*kissr* genes has been found in the brain and gonads of investigated species, as in turbot, demonstrating its conserved expression in the CNS along with its putative role in fish reproduction. It is worth mentioning that, in the present study, *kiss1* was more widely expressed in different tissues than *kiss2*. The *kissr* mRNA was also expressed in the eye, and *kiss1* mRNA was expressed in the intestine, which was in line with previous studies, suggesting that the Kiss/Kissr system might have additional roles in turbot beyond reproduction (14, 20). In addition, differences in expression levels of *kiss*/*kissr* among different brain regions were found in turbot. Expression levels of *kiss1* were highest in the Me and Hy, and *kiss2* was highest in the Hy, which was consistent with their expression patterns in adults of many other fish, including medaka, European sea bass, striped bass (*Morone saxatilis*), and Nile tilapia, among others (16, 60, 66). While turbot *kissr2* and *kissr3* had wide distributions, and *kissr3* had wider distribution than *kissr2*. A similarly wide distribution was observed in the medaka brain (17). However, the opposite was true in zebrafish and European seabass, with *kissr2* showing a wider distribution than *kissr3* (61, 67). These data suggest that the Kiss/Kissr system has diverse functions in the brain.

Numerous previous studies have used different approaches to help clarify the reproductive function of the Kiss/Kissr system, which has been established as an important regulator in the HPG axis of mammals and teleosts (10, 11, 68). The relative expression profiles of multiple genes in the Kiss/Kissr system in the brain and gonad were analyzed in different gonad developmental stages in the present study. First, the expression profiles of the four *kiss*/*kissr* genes were similar in the brains of both sexes, where the expression of the *kiss*/*kissr* genes gradually decreased from the immature stage to the mature stage, and increased again during the postspawning period, with the same result in the Me and Hy. The *kiss*/*kissr* genes can be different during the gonadal stages between males and females in different fish species (24, 30, 31, 68). For example, *kiss1* levels continuously declined from the immature to postspawning stages in male chub mackerel, but its level in females showed no significant difference during ovarian development (24). The expression of *kiss2* in the brain of seahorses significantly declined at the pregnancy stage. In fathead minnow (*Pimephales promelas*) the expression of *kissr2* mRNA was low in the advanced gonadal stages in males (69). In addition, the expression profiles of four *kiss*/*kissr* genes in the testis and ovaries of turbot showed different expression profiles. Studies in the Senegalese sole revealed the genes of the Kiss/Kissr system were expressed in all germ cell types during spermatogenesis (63). Moreover, in sea bass, expressions of *kissr2* and *kissr3* peaked at the initiation and completion of the spermiation period (21), which was consistent with the *kissr* expression patterns in male turbot. These results in the brain and gonad of both male and female turbot suggest the positive involvement of the Kiss/Kissr system in turbot reproduction cycle, as in other fishes and mammals. Moreover, the Kiss/Kissr system is essential in other reproductive activity. Kiss2 is involved in sex differentiation in chub mackerel and pejerrey (30, 65). Kiss1 and Kiss2 is essential for male spermiation in the striped bass (66).

It is well known that seasonal fish reproduction is modulated by a wide variety of environmental factors. Photoperiod is generally thought to synchronize sexual maturation and determine spawning time through the activation of neuroendocrine pathways in temperate regions (48, 49). The discovery of the Kiss/Kissr system has provided important insights into the relationship between seasonal reproduction and photoperiod. In mammals, studies have revealed that the Kiss/Kissr system is the most potent activator of GnRH neurons, and the kisspeptin neurons project directly to GnRH neurons and *kissr* is located at the key site of GnRH neurons (59, 67, 70). Evidence in sheep has suggested that *kiss* neurons in the arcuate nucleus could directly innervate GnRH neuron somata and dendrites to mediate the induction of *lhβ* and *fhsβ* gene expression (71). The *in vivo* action of Kiss also investigated in some fish species. In lined seahorse, the injection of Kiss2-10 significantly increased *fhsβ* and *lhβ* at 6h post-injection and it was higher than 3h in the expression of *fhsβ* (31). Significant increases in *fhsβ* and *lhβ* mRNA levels were seen in the pituitary of zebrafish injected with Kiss2 at 12h after injection (15, 47). Treatment with 0.1 and 0.5 μg/g of Kiss significantly increased the GTH mRNA levels in the pituitary of female and male cinnamon clownfish at 2, 4 and 6 weeks after injection (42). Moreover, in goldfish, compared to 0.01μg/g, the high doses of 0.1 and 1.0 μg/g significantly increased serum LH levels after 6 h, indicating a dose-dependent manner (19). Therefore, it is concluded that the high dose increased relative mRNA levels (*fhsβ*, *lhβ* and *GTH*) at detection time. In the present study, Kiss1-10 and Kiss2-10 stimulated the expression of *lhβ*, *fhsβ*, and *gthα* in the pituitary of turbot, indicating that the Kiss/Kissr system could elevate GTH release in turbot, which is conserved among vertebrates (31). Different from previous studies was that the expression of *fhsβ* and *gthα* mRNA at high dose of 1000ng/g after 3h injection was lower than 100ng/g after 3h injection. It was proposed that the Kiss directly increased the expression of *lhβ*, *fhsβ*, and *gthα* in pituitary cells, but the effects depended on the time course and dose. And the investigation of an extended period in the Kiss to genes in pituitary cells would be done in future.

On the other hand, GnRH neurons projecting to the pineal gland, which is a key gland in transducing light signals in the circadian production of melatonin, provide support for the role of GnRH in the transduction of seasonal photoperiod changes (49). Taken together, these observations show the importance of the Kiss/Kissr system in the mechanisms regulating seasonal reproduction. Furthermore, recent studies in mammals and in seasonal breeders of fish have demonstrated that reproduction can be controlled *via* the Kiss/Kissr system. In European sea bass, manipulating the photoperiod affected the expression of *kiss1* and *gnrh2* in the forebrain-midbrain to activate the reproductive axis (49). Increased *kiss2* and *kissr2* gene expression in Atlantic cod also showed the potential role of the Kiss/Kissr system in the entrainment of reproduction (49, 62). Similarly, changing the photoperiod in the present study directly regulated the seasonal gonadal development and modulated the expression of the *kiss/kissr* genes. However, further investigation is needed to better characterize the mechanism by which the Kiss/Kissr system affects reproduction in turbot.

In conclusion, the present study investigated the Kiss/Kissr system and the changes in the expression of *kiss* and *kissr* genes during the reproductive cycle in turbot. All four highly conserved kisspeptin genes (*kiss1*, *kiss2*, *kissr2*, and *kissr3*) of the Kiss/Kissr system were present in this species, which was different from other Pleuronectiformes species. The expression levels of *kiss* and *kissr* in HPG during the reproductive stages and in the pituitary gland after administration of synthetic Kiss1-10 and Kiss2-10 suggest the positive involvement of the Kiss/Kissr system in controlling the seasonal reproductive cycle in turbot.

## Data Availability Statement

The original contributions presented in the study are included in the article/**Supplementary Material**. Further inquiries can be directed to the corresponding author.

## Ethics Statement

The animal study was reviewed and approved by Animal Research and Ethics Committees of Qingdao Agricultural University.

## Author Contributions

CZ: Performed experiments, analyzed the data, wrote, and approved the manuscript; BW: Performed experiments, analyzed the data, edited and approved the manuscript; YL, CF, SX, WW, and QL: Performed experiments and reviewed the manuscript; JL: Designed research, edited and approved the manuscript. All authors contributed to the article and approved the submitted version.

## Funding

This work was supported by National Natural Science Foundation of China (31802319, 32072949 and 31702321), Advanced Talents Foundation of QAU (6631119055), Key Special Project for Introduced Talents Team of Southern Marine Science and Engineering Guangdong Laboratory (Guangzhou) (GML2019ZD0402), China Agriculture Research System (CARS-47).

## Conflict of Interest

The authors declare that the research was conducted in the absence of any commercial or financial relationships that could be construed as a potential conflict of interest.

## Publisher’s Note

All claims expressed in this article are solely those of the authors and do not necessarily represent those of their affiliated organizations, or those of the publisher, the editors and the reviewers. Any product that may be evaluated in this article, or claim that may be made by its manufacturer, is not guaranteed or endorsed by the publisher.
