# Supplementary material for: New Evidence for the Existence of Two Kiss/Kissr Systems in a Flatfish Species, the Turbot (Scophthalmus maximus), and Stimulatory Effects on Gonadotropin Gene Expression
Source: Front Endocrinol (Lausanne). 2022 Jun 15;13:883608. doi: 10.3389/fendo.2022.883608 (PMC9240279; doi:10.3389/fendo.2022.883608)
Supplement: Supplementary file 1 [file Image_1.pdf]

Supplementary FIGURE 1

A turbot *kiss1*

```
1     ACAT GGG GAC AGG GGA TCT CAG TCA GCA CTC GCC TGC AGC GTA AAG   46
47     AGG AAT CTC AGC AGC TAC AAC AAA ATG ACT GTC ACC ATG CTA CGA   91
                                                     M   L   R
92     CTC GTT GCC GTG ATG ATT GCT GCT TTG TCA ACC GAC GTC TGC ACG   136
5       L   V   A   V   M   I   A   A   L   S   T   D   V   C   T    19
                     Signal peptide
137    TCC AGC AGT TTG AAG TCC ATG CAC AAC AGT GAA GAT GAG GGA ATA   181
20      S   S   S   L   K   S   M   H   N   S   E   D   E   G   I    34
182    CTC AAA GCC TTG AGA GAT TTA ACC GAT GCC GCA ATG GCA ACG TCA   226
35      L   K   A   L   R   D   L   T   D   A   A   M   A   T   S    49
227    GCA AAG AAT TCA TGG AAC TTT CCT GCT GAT AAG ATC CAT TCA TCT   271
50      A   K   N   S   W   N   F   P   A   D   K   I   H   S   S    64
272    GAT GGA AAG TTT CCC AGA TCA GGA TGG TGG ATC TCA AAG GTG ATC   316
65      D   G   K   F   P   R   S   G   W   W   I   S   K   V   I    79
317    TTC CCT CAG ACC ATC AAG AAG CAT CAA GAT GTG TCT TCA TAC AAC   361
80      F   P   Q   T   I   K   K   H   Q   D   V   S   S   Y   N    94
362    CTC AAC TCC TTT GGT CTC CGT TAT GGA AAA TGA CAC AAG AAC TTA   406
95      L   N   S   F   G   L   R   Y   G   K   *                    105
                 Kiss1 peptide
407    TGT ATA TTC TTA TTG TGT GGG GTG TGT AAC TTT GAA TTA TAG TAT   451
452    TCT TAT TCA ATT TGT ATA TTC AGA GTG AGG TAC CAA AAC AAC TGT   496
497    CAC TGT TAA AAA CGC AAT AAA AAA ACT CAG TTG AAA ATT AAA AAA   541
542    AAA AAA GAA AAA AAA AAA CAA AAA AAA AAA AAA AAA AAA           580
```

B turbot *kiss2*

```
1      CTG CGG AGT GGA TGA ACG TTA ACA GGC ACA GGG AGG ATC TGA ATC   45
46     TTG TGT TCA AAG ATG AGA CTT GTG GCT CTG GTT GTG GTG TGT GGC   90
                        M   R   L   V   A   L   V   V   V   C   G
91     CTG ATC GTC AGT CAG GAT GGA CGG AGC ACA GGA GCA GCT CTG CCA   135
13      L   I   V   S   Q   D   G   R   S   T   G   A   A   L   P    27
            Signal peptide
136    GGA TAT GAC TCG ACA CAA GGG ACA CGA GCA ACA GGG TCC GTC CTC   180
28      G   Y   D   S   T   Q   G   T   R   A   T   G   S   V   L    42
181    TCC GCG CTC AGG AGG AGG ACC GCG GGA GAT CCG CTG GCG GAG GAC   225
43      S   A   L   R   R   R   T   A   G   D   P   L   A   E   D    57
226    CCC GGC CTG TGC TTC TCC CTT AGG CAA AAC GAG GAG CAG CTG CAG   270
58      P   G   L   C   F   S   L   R   Q   N   E   E   Q   L   Q    72
271    CTG CTG TGC AAC GAC CGC AGG AGC AAA TTC AAC TTC AAC CCG TTT   315
73      L   L   C   N   D   R   R   S   K   F   N   F   N   P   F    87
316    GGC CTT CGC TTC GGG AAA CGC TAC ATC TAC AGG AGA GCC GTT ACA   360
88      G   L   R   F   G   K   R   Y   I   Y   R   R   A   V   T    102
         Kiss2 peptide
361    AGA GCC AGG ACG AAT AAG TTC TCG CCC CTT TCT CTC TTC CCG CGA   405
103     R   A   R   T   N   K   F   S   P   L   S   L   F   P   R    117
406    GAA TTA GAG GTG CCC ACC TGA GAA TTA GTC TTT TCC ATA GGT GGT   450
118     E   L   E   V   P   T   *                                    124
451    TCA CAA GGC AGC ATC GTG TTT TCC TGA ATA ACC CCT TTT TTT TTT   495
496    ACA CCT AAC TTC ACT GTG AAA TGA CTT TGA AAT CTA CAA CAA TTC   540
541    AAA AGT TAC GAG AGA AAA TAA ACG ACC AAA AAA AAA AAA AAA AAA   585
586    AAA AAA AAA AAA                                               597
```

Supplementary FIGURE 1 cDNA and deduced amino acid sequence of turbot *kiss1* (**A**) and *kiss2* (**B**). The putative signal peptide is underlined. The mature peptide, Kiss1-10 and Kiss2-10, are boxed. The different amino acids between Kiss1-10 and Kiss2-10 indicated in red. The stop codon is indicated by an asterisk. The potential polyadenylation signals (AATAAA) are in bold.
