# Supplementary material for: New Evidence for the Existence of Two Kiss/Kissr Systems in a Flatfish Species, the Turbot (Scophthalmus maximus), and Stimulatory Effects on Gonadotropin Gene Expression
Source: Front Endocrinol (Lausanne). 2022 Jun 15;13:883608. doi: 10.3389/fendo.2022.883608 (PMC9240279; doi:10.3389/fendo.2022.883608)
Supplement: Supplementary file 2 [file Image_2.pdf]

Supplementary FIGURE 2

A

Turbot kiss1

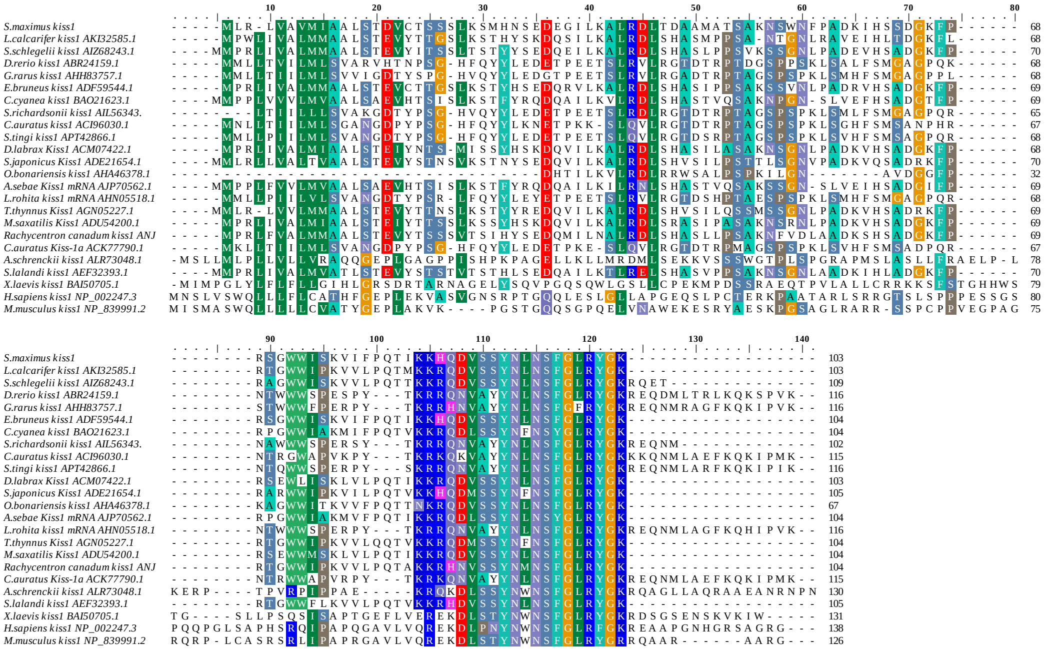

B

Turbot kiss2

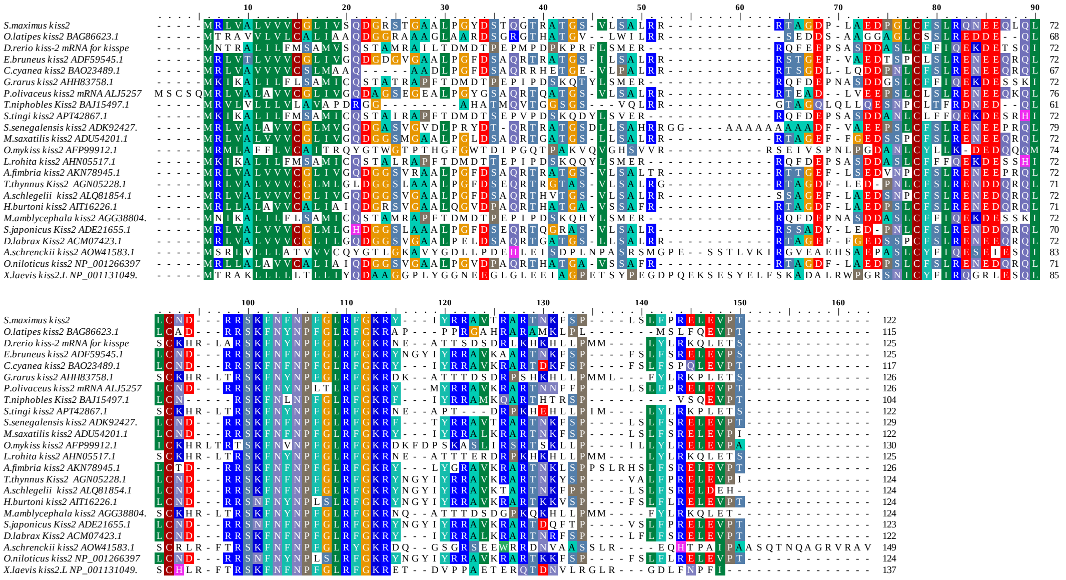

Supplementary FIGURE 2

Alignment of the deduced amino acid sequences for kiss1 and kiss2 from turbot and several teleost species. (A) kiss1, (B) kiss2.
