# Supplementary material for: New Evidence for the Existence of Two Kiss/Kissr Systems in a Flatfish Species, the Turbot (Scophthalmus maximus), and Stimulatory Effects on Gonadotropin Gene Expression
Source: Front Endocrinol (Lausanne). 2022 Jun 15;13:883608. doi: 10.3389/fendo.2022.883608 (PMC9240279; doi:10.3389/fendo.2022.883608)
Supplement: Supplementary file 3 [file Image_3.pdf]

Supplementary FIGURE 3

A

Turbot kissr3

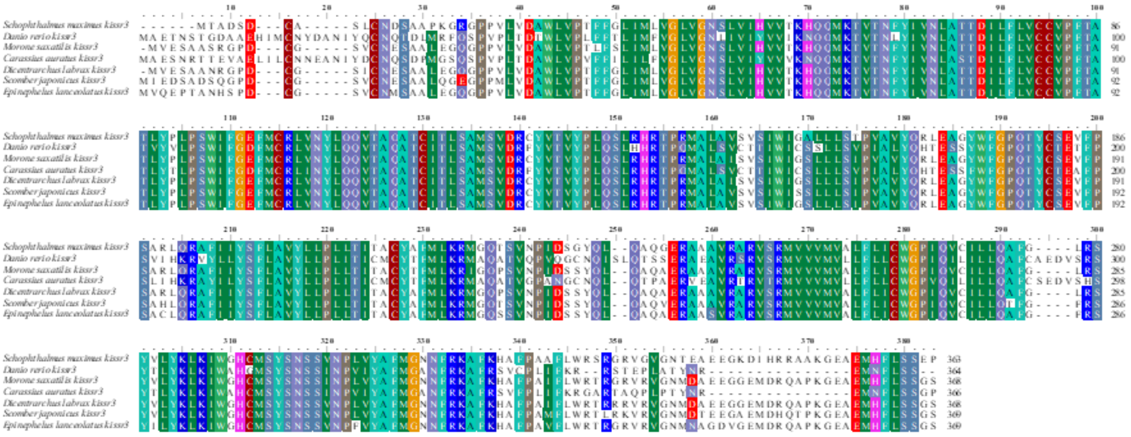

B

Turbot kissr2

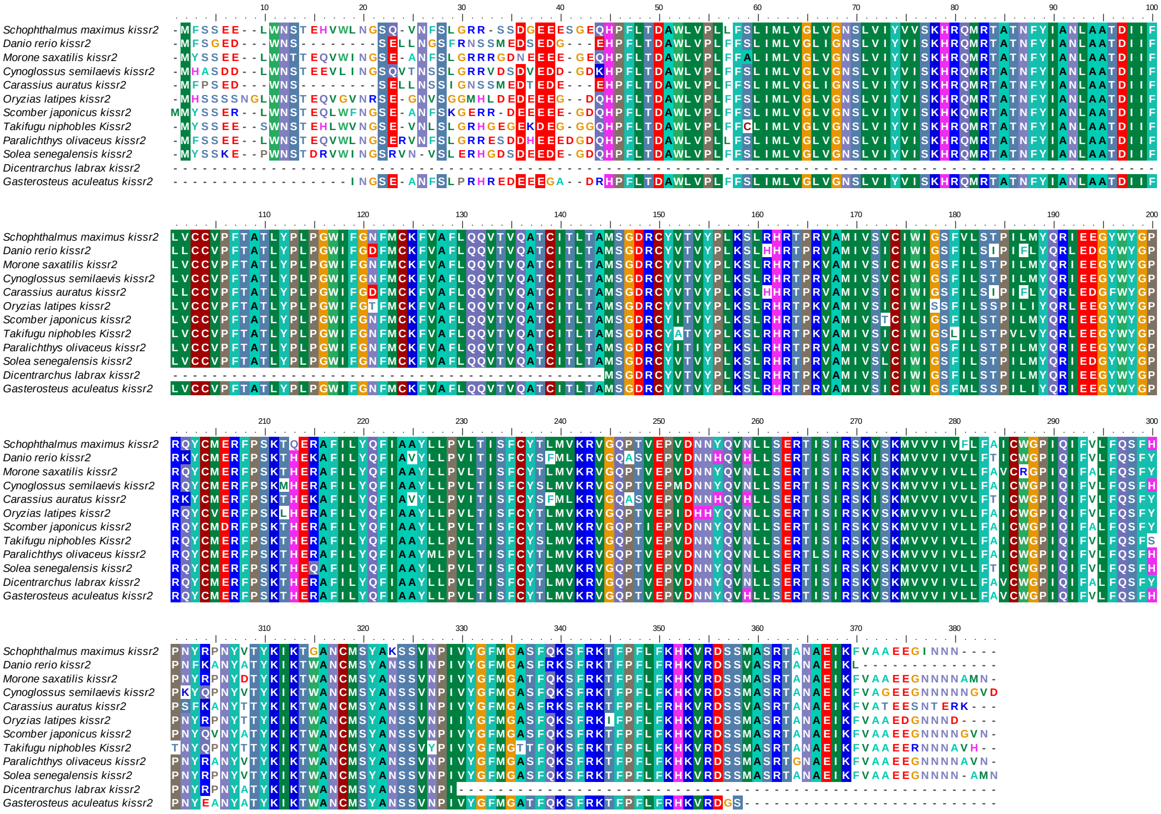

Supplementary FIGURE 3 Alignment of the deduced amino acid sequences for kissr3 and kissr2 from turbot and several teleost species. (A) kissr3, (B) kissr2.
