# Supplementary material for: New Evidence for the Existence of Two Kiss/Kissr Systems in a Flatfish Species, the Turbot (Scophthalmus maximus), and Stimulatory Effects on Gonadotropin Gene Expression
Source: Front Endocrinol (Lausanne). 2022 Jun 15;13:883608. doi: 10.3389/fendo.2022.883608 (PMC9240279; doi:10.3389/fendo.2022.883608)
Supplement: Supplementary file 4 [file Image_4.pdf]

Supplementary FIGURE 4

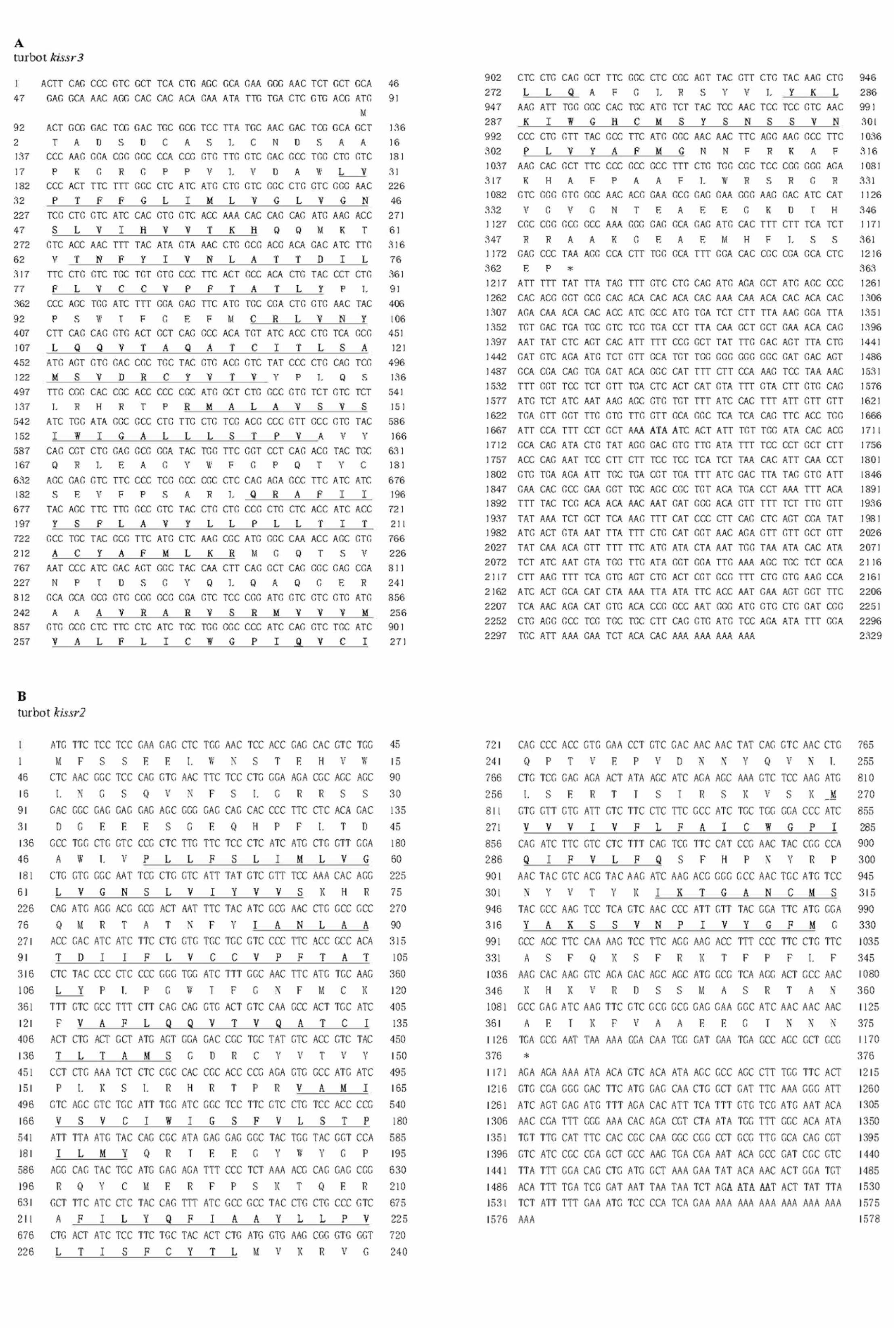

Supplementary FIGURE 4 cDNA and deduced amino acid sequence of turbot *kissr3* (**A**) and *kissr2* (**B**). Seven putative hydrophobic transmembrane (TM) domains are underlined. The stop codon is indicated by an asterisk. The potential polyadenylation signals (AATAAA) are in bold.
